# Supplementary material for: Millennial-timescale thermogenic CO2 release preceding the Paleocene-Eocene Thermal Maximum
Source: Nat Commun. 2025 Jun 30;16:5375. doi: 10.1038/s41467-025-60939-3 (PMC12209464; doi:10.1038/s41467-025-60939-3)
Supplement: Supplementary file 1 — Supplementary Information [file 41467_2025_60939_MOESM1_ESM.pdf]

Supplementary information for

Millennial-timescale thermogenic CO_2 release preceding the Paleocene-Eocene Thermal Maximum

Shijun Jiang[1,2,*], Ying Cui[3,*], Yasu Wang[1], Maura De Palma[3], B. David A. Naafs[4], Jingxin Jiang[5], Xiumian Hu[5], Huaichun Wu[6], Runjian Chu[6], Yangguang Gu[7], Jiuyuan Wang[8], Yizhou Huang[4], Miquela Ingalls[9], Timothy J. Bralower[9], Shiling Yang[10], James C. Zachos[11], and Andy Ridgwell[12]

[1]State Key Laboratory of Marine Resource Utilization in South China Sea, Hainan University, Haikou 570228, China

[2]Southern Marine Science and Engineering Guangdong Laboratory (Zhuhai), Zhuhai 519082, China

[3]Department of Earth and Environmental Studies, Montclair State University, Montclair, NJ 07043, USA

[4]Organic Chemistry Unit, School of Chemistry and School of Earth Sciences, University of Bristol, Bristol, UK

[5]State Key Laboratory of Critical Earth Material Cycling and Mineral Deposits, School of Earth Sciences and Engineering, Nanjing University, Nanjing 210023, China

[6]School of Ocean Sciences, China University of Geosciences (Beijing), Beijing 100083, China

[7]South China Sea Fisheries Research Institute, Chinese Academy of Fishery Sciences, Guangzhou, China

[8]SKLab-DeepMinE, MOEKLab-OBCE, School of Earth and Space Sciences, Peking University, China

[9]Department of Geosciences, The Pennsylvania State University, University Park, PA 16802, USA

[10]Key Laboratory of Cenozoic Geology and Environment, Institute of Geology and Geophysics, Chinese Academy of Sciences, PO Box 9825, Beijing 100029, China

[11]Department of Earth and Planetary Sciences, University of California, Santa Cruz, CA 95064, USA

[12]Department of Earth and Planetary Sciences, University of California, Riverside, CA 92521, USA

*Correspondence should be addressed to: cuiy@montclair.edu (Y. Cui) and ssj0047@my.fsu.edu (S. Jiang).

Contents

1. Regional geology

2. Assessment of diagenetic effects on $\delta^{18}O_{carb}$

3. Age model option 2 and sedimentation rate

4. Sediment composition control on mercury (Hg) records at the study site

5. cGENIE Earth system modeling of the POE

Supplementary References

Supplementary Tables S1

Supplementary Figures S1-S9

Supplementary Text

1. Regional geology

The Kuzigongsu section (39°45'10" N, 75°17'29" E) lies in the Tarim basin (37°10'–42°00' N, 75°00'–93°00' E), a northwestward rhombic-shaped depression (563,000 km^2) that is bordered by the Tianshan Mountains to the north and the West Kunlun Mountains to the west, and the Altyn Tagh Mountains to the southeast[1] (Fig. 1). The pre-Sinian basement of the Tarim basin is composed of highly metamorphosed Archean and Proterozoic crystalline rocks, which mostly crop out around the edge of the basin; and the sedimentary infill mainly consists of late Proterozoic through Cenozoic marine and terrestrial sediments[1, 2, 3, 4, 5, 6]. The Paleozoic sediments are widespread and primarily consist of platform carbonates and fine-grained clastics[7], whereas coarse-grained fluvial and lacustrine sequences are dominant during the Mesozoic and Cenozoic[5, 8], with multiple episodes of conglomerate deposition reflecting contraction and uplift of the basin margins[9].

Marine deposition in the Tarim Basin initiated in the Late Cretaceous and occurred intermittently through the Paleogene[10, 11, 12, 13]. During these periods, the Tarim Basin was a shallow epicontinental sea connecting the central Eurasia (i.e., the eastern Tethys) with the western Mediterranean Tethys[10, 14, 15], and is believed to have acted as the major source of moisture and the driver of land-sea thermal exchange in the Asian interior and Asian monsoon system[16, 17, 18]. The sea retreated westward after five successive eastward marine transgressions through the present-day Alai Valley, eventually separated from the western Tethys and the Arctic Sea during latest Eocene to early Oligocene (Figs. 1 and S1) and became a remnant sea called Paratethys[10, 11, 19]. The largest transgression occurred near the Paleocene-Eocene boundary when the eastern Tethys was a restricted shallow water carbonate platform[20], which is well documented

in the Qimugen Formation in the southwestern Tarim Basin. The Kuzigongsu section was originally deposited along an east-facing terrigenous-rich foreland basin referred to as the "Tarim Sea" gulf in the eastern Tethys, which was connected to the Siberian Sea to the north[21, 22].

The study section is 47.6 m thick and spans the lower part of the Qimugen Formation, which is primarily composed of limestone, marlstone (or calcareous mudstone), and bivalve-rich limestone (Fig. 1). The base of the section is a 4 m-thick grey massive limestone, which is overlain by alternate grayish-green calcareous mudstones and grey marlstones with a distinct oyster limestone in the middle and massive limestone on the top (Figs. S1). During sampling, the top >1 m of surface weathered rock was removed, and fresh rock chips were carefully picked to avoid weathering contamination.

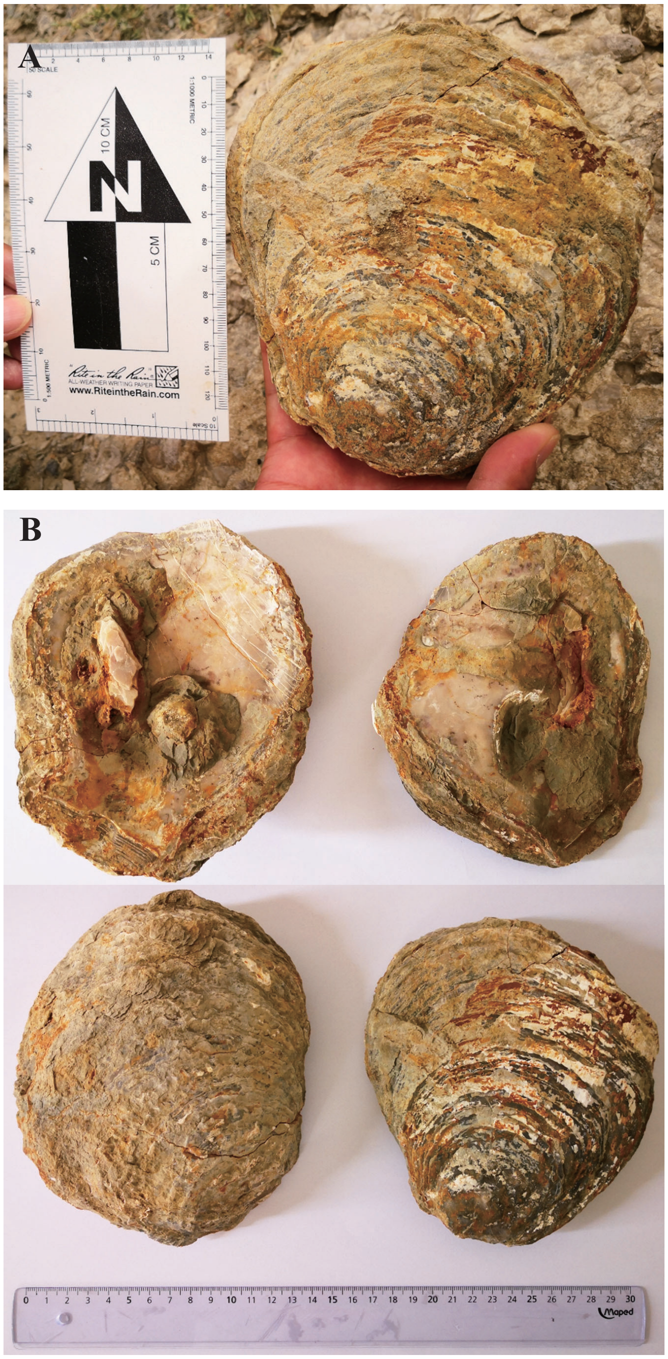

Figure S1. Oyster fossil (A, B–*Crassostrea* spp.) from 29.8 m depth used for clumped isotopes.

2. Assessment of diagenetic effects on $\delta^{18}O_{carb}$

The strong correlation between $\delta^{18}O_{carb}$ and $\delta^{13}C_{carb}$ during the POE and the PETM (R^2 = 0.69 and 0.87, respectively; Fig. S2) suggests that $\delta^{18}O_{carb}$ cannot be used to faithfully

reconstruct the sea surface temperature at the study site. However, diagenetic alteration of $\delta^{18}O_{carb}$ outside of the POE and PETM interval is considered to be limited based on the following: (1) micrites dominate the matrix of all mudstone and wackestone samples with few visible secondary minerals such as calcite, dolomite, and siderite[20]; (2) weak correlation between $\delta^{13}C_{carb}$ and $\delta^{18}O_{carb}$ for samples outside the PETM and POE intervals ($R^2 = 0.03$, $n = 332$; Fig. S2); (3) the low to moderate Mn/Sr values (ranging from 0.04 to 5.4 with an average of 2.44) and weak correlation between Mn/Sr and $\delta^{18}O_{carb}$ ($R^2 = 0.04$, Fig. S2); and (4) most samples fall within the Cenozoic carbonate "lithification" zone (Fig. S2)[23], suggesting limited post-depositional alteration (Fig. S2).

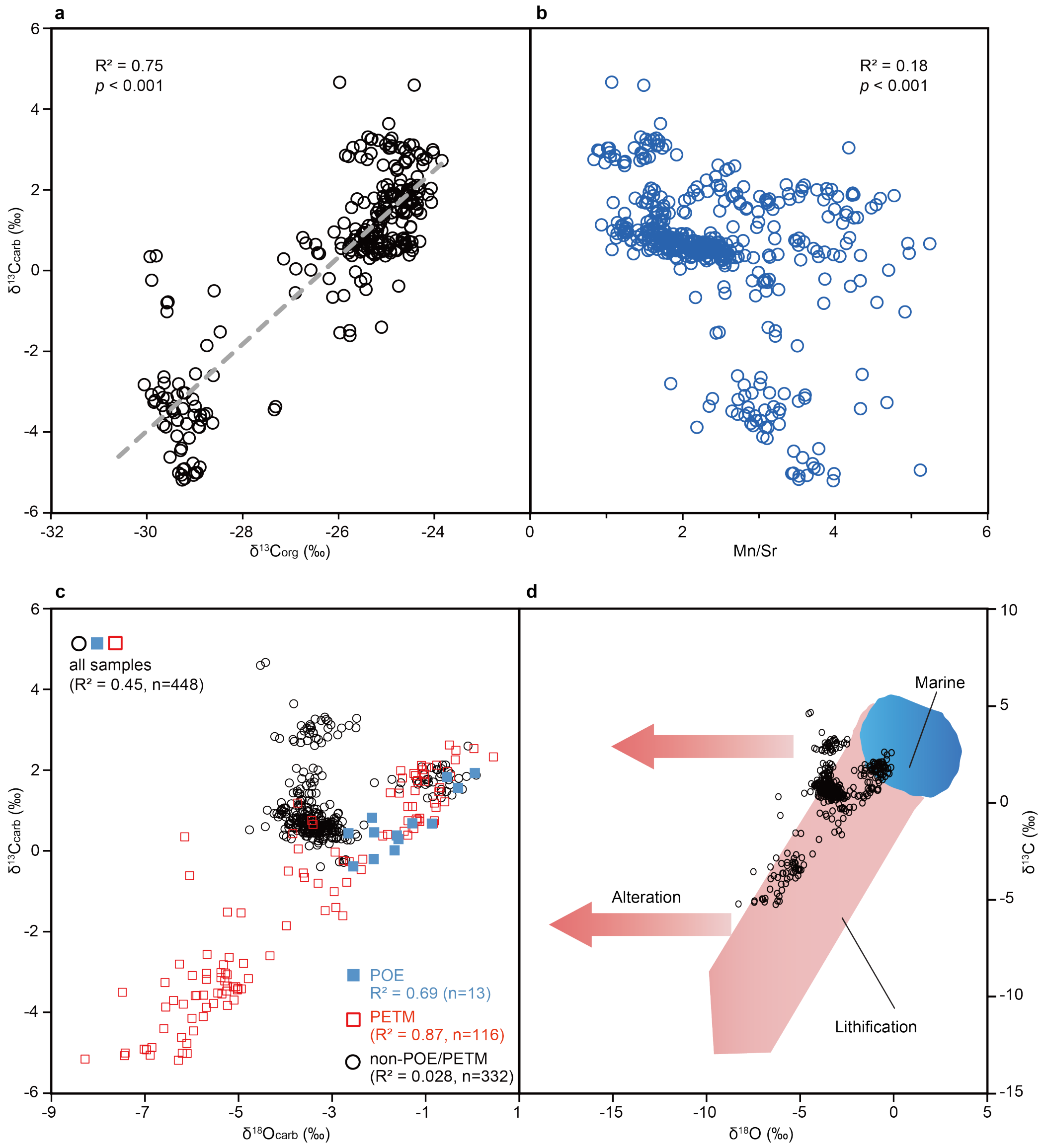

Figure S2. Assessment of diagenetic impact on samples used in this study. Cross plot of bulk carbonate $\delta^{13}C_{carb}$ vs. $\delta^{13}C_{org}$ (**a**) and Mn/Sr (**b**), and $\delta^{13}C_{carb}$ vs. $\delta^{18}O_{carb}$ (**c**) for all samples, non-POE and -PETM samples (black circles), POE samples (blue filled squares), and PETM samples (red squares). Note the poor correlation for those samples outside the POE and PETM intervals, and the strong correlation for the POE and PETM samples. (**d**) projection of all samples (black circles) on Cenozoic primary limestones/dolostones[23]. Blue area is dominantly marine pore fluids, red area contains significant meteoric water component, and both areas define the 'lithification' zone. Post-depositional deep burial and/or metamorphic alteration yield data to the left of the lithification domain (red arrows).

3. Age model option 2 and sedimentation rate

The PETM interval at the study section is associated with abrupt changes in lithologic facies from tidal flat to basin and in sedimentation rates based on microfacies and biostratigraphic analyses[20, 24]. To account for a possible depositional hiatus at about 30-m depth reflected by lithological changes, two age model options are provided. Age model option 1 assumes a relatively complete PETM record and age model option 2 considers possible truncation at ~30 m depth where a change in lithology from mudstone to bioclastic limestone coincides with an abrupt increase in $\delta^{13}C_{carb}$ and wt.% $CaCO_3$ is observed (Fig. 2). Under this assumption, the 6–10 m cycles shown by spectral analysis are interpreted as ~20 kyr precessional cycles (Fig. S3). The filtered MS data suggest that the Kuzigongsu section only contains ~3 precession cycles (a total duration of ~60 kyr) of the PETM record, which indicate that the recovery phase of the PETM at the Kuzigongsu section may be truncated at ~30 m. The estimated sedimentation rate based on age model option 2 is 4–30 cm kyr^{-1}.

A range of feasible sedimentation rates is required to construct astronomically-tuned age model. The duration of the CIE during PETM was estimated to be 100–200 kyr based on astrochronology[25, 26, 27, 28] and ^3He age models[29, 30]. Using a CIE duration of 100–200 kyr and a PETM thickness of 10.6 m at the study site, the calculated sedimentation rate ranges from 5.3 to 10.6 cm kyr^{-1}, therefore, 4 to 12 cm kyr^{-1} was set as the range of reasonable sedimentation rates for time scale optimization (TimeOpt; Meyers[31]) and correlation coefficient (COCO; Li et al.[32]) analyses (Figs. S3 to S5).

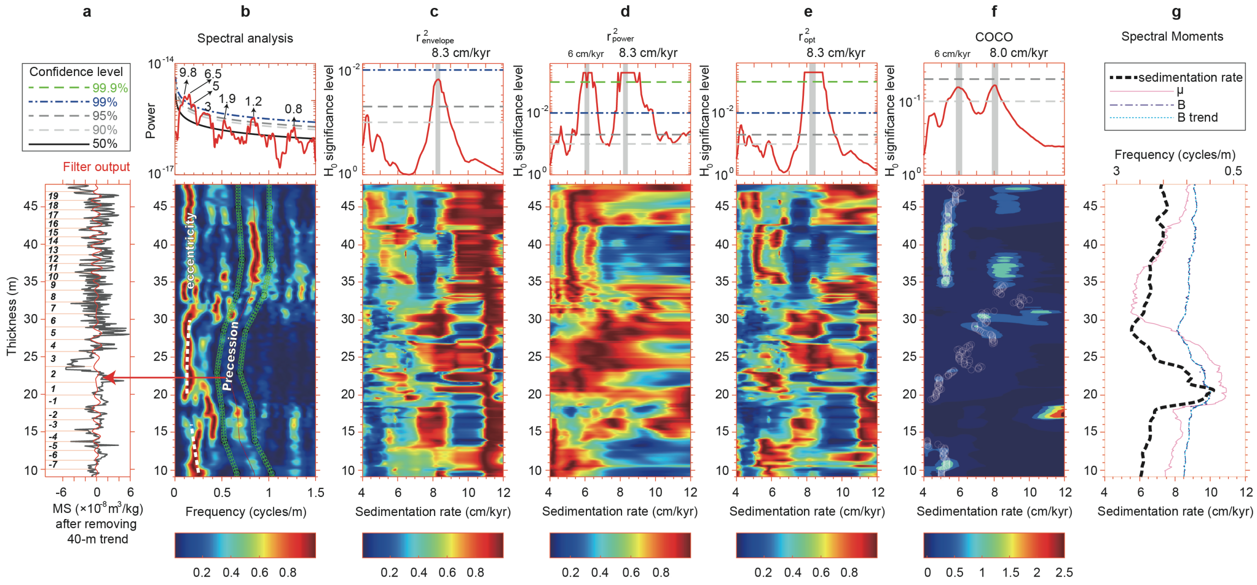

Figure S3. Cyclostratigraphy of the Kuzigongsu section. **a**. Detrended magnetic susceptibility (MS) showing dynamic filtered 1.2 m & 1.9 m cycles. **b**. Multi-Taper Method (MTM) Spectra (2π tapers) and evolutionary power spectra. 2π MTM (top) are shown with mean, 90%, 95% and 99% confidence level. Evolutionary power spectra (bottom, color areas) are calculated using a 10 m window. **c-e**. TimeOpt analysis and evolutionary TimeOpt (eTimeOpt) sedimentation rate map of the MS series. The TimeOpt (r^2_{envlop}, r^2_{power}, r^2_{opt}, top) are shown with 90%, 95%, 99% and 99.9% confidence level (2,000 Monte Carlo simulations). The eTimeOpt (bottom, color areas) are calculated using a 10 m window. **f**. COCO analysis and evolutionary COCO (eCOCO) sedimentation rate map of the MS series. The COCO (top) are shown with 90% and 95% confidence level (2,000 Monte Carlo simulations). The eCOCO (bottom, color areas) are calculated using a 10 m window. For both the (e)TimeOpt and (e)COCO analyses, tested sedimentation rates range from 4 to 12 cm kyr^{-1} with a step of 0.1 cm kyr^{-1}. **g**. Spectral Moments analysis for the MS series. The bandwidth (B), mean frequency (μ_f) and the estimated sedimentation rates using the mean sedimentation rate of 7 cm kyr^{-1}.

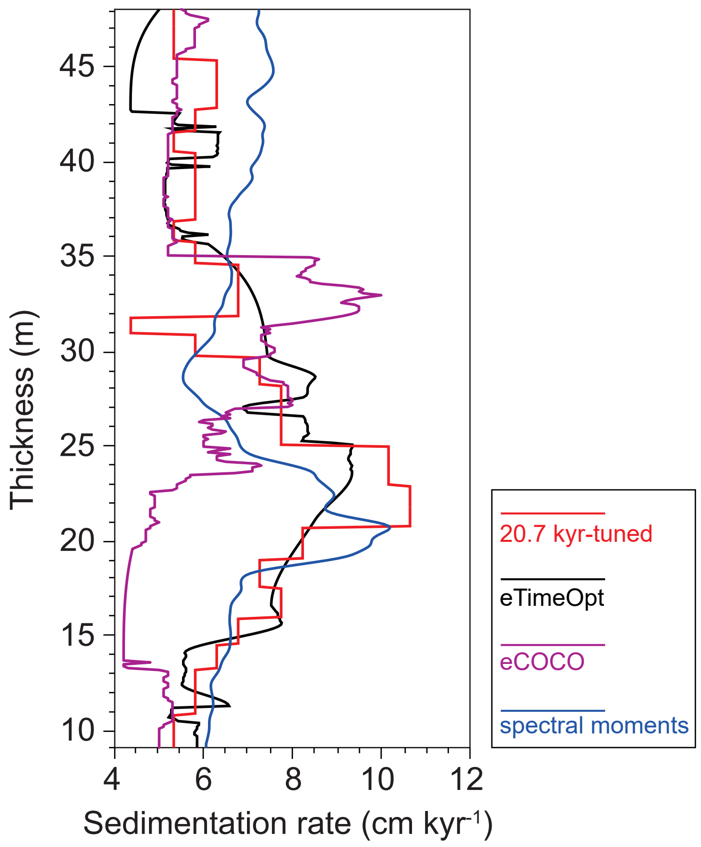

Figure S4. Sedimentation rate estimated based on four independent methods. These include the tuning of filtered cycles to match the precession period of 20.7 kyr (red line), evolutionary TimeOpt method (eTimeOpt; black line), evolutionary correlation coefficient method (eCOCO; purple line), and spectral moments (blue line). Our age model option 1 is associated with the sedimentation rate estimated based on the 20.7 kyr precession cycle tuning.

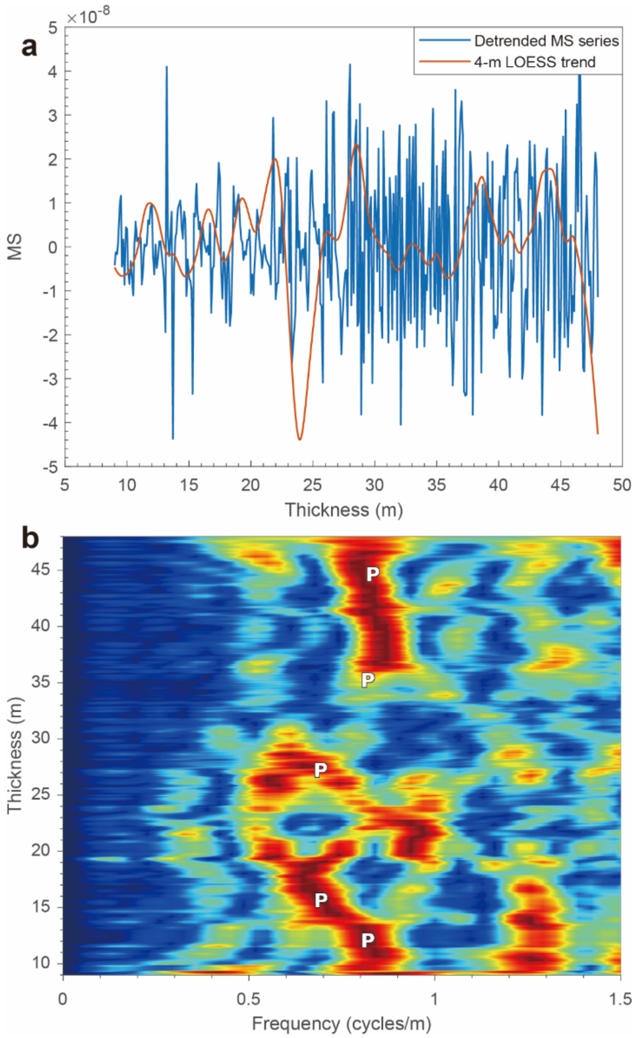

Figure S5. Detrended magnetic susceptibility (MS) values and evolutive harmonic analysis (EHA). (a) Detrended MS values to avoid possible/potential influence of eccentricity cycles. (b) EHA spectrogram of the detrended MS series. Sliding window is 8 m with a step of 0.1 m.

4. Sediment composition control on mercury (Hg) records at the study site

In order to explore the potential impact of sediment composition on Hg concentration at the study site, we explore the relationship between Hg concentration, TOC and wt.% $CaCO_3$ (Fig. S6). The slope of Hg concentrations vs. TOC is steeper for samples within the POE intervals than those during the PETM and other intervals, which suggests excess Hg in these samples, similar to those observed by Kender et al.[33] during the PETM (Fig. S6a). The weak

correlation between wt.% $CaCO_3$ and Hg concentrations ($R^2 = 0.15$, Fig. S6b) suggests that the Hg concentrations are not sensitive to the factors controlling wt.% $CaCO_3$, such as carbonate production, dissolution, burial, and mixing of siliciclastic inputs (Fig. S6b).

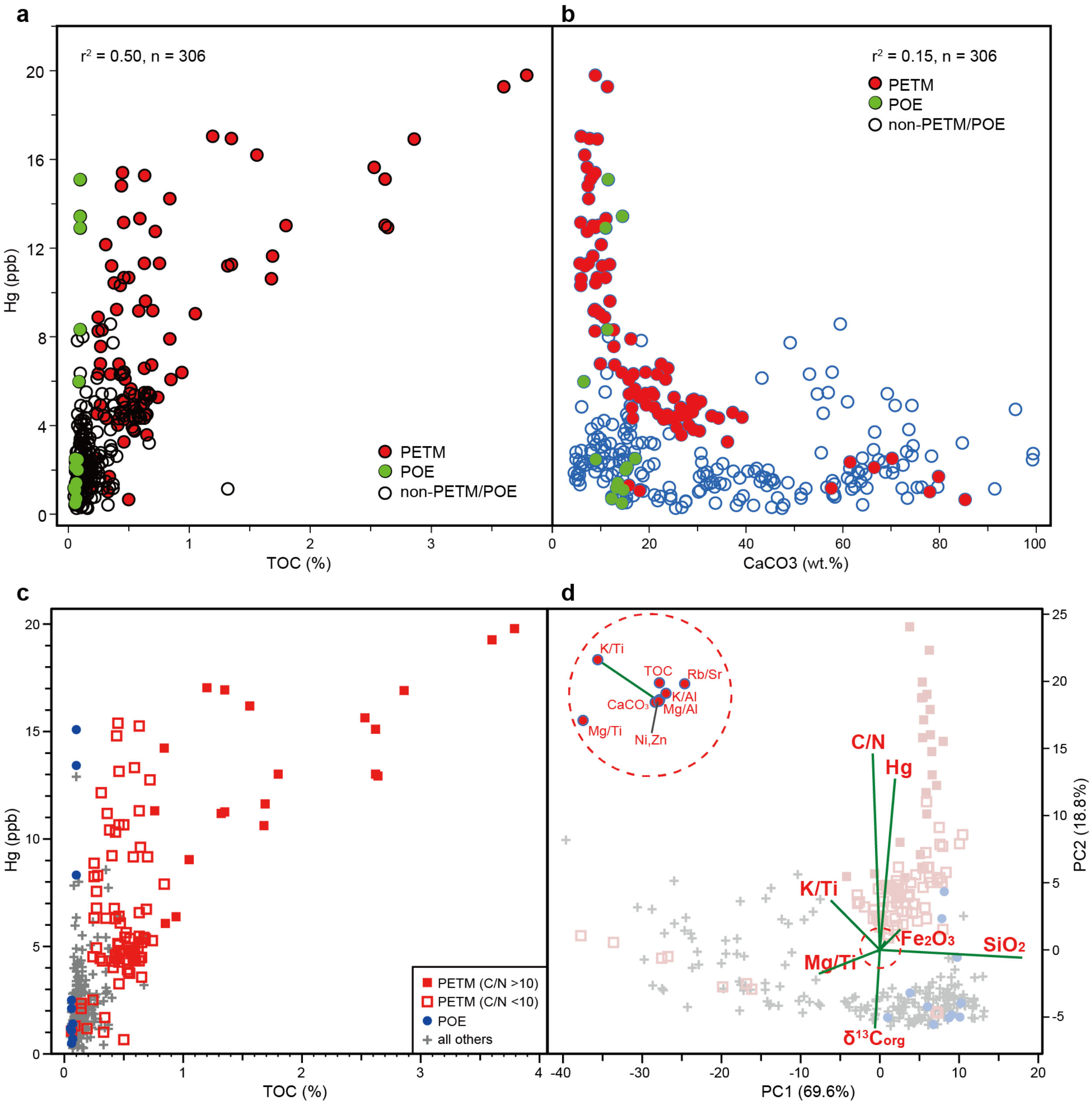

Figure S6. Relationship between mercury (Hg) and total organic carbon (TOC), carbonate content ($CaCO_3$ wt.%) and principal component analysis (PCA). Cross plot of Hg concentration with TOC (**a**) and $CaCO_3$ (wt.%) (**b**). PETM samples (red circles), POE (green circles), samples outside of PETM and POE (black circles). (**c**) Cross plot of Hg concentration (ppb) with TOC

(%) (**d**) PCA results that show the relative associations of sedimentary Hg with C/N ratios, $\delta^{13}C_{org}$ and other geochemical proxies.

5. cGENIE Earth system modeling of the POE

5.1 Paired "pH- δ^{13}C" Forcings

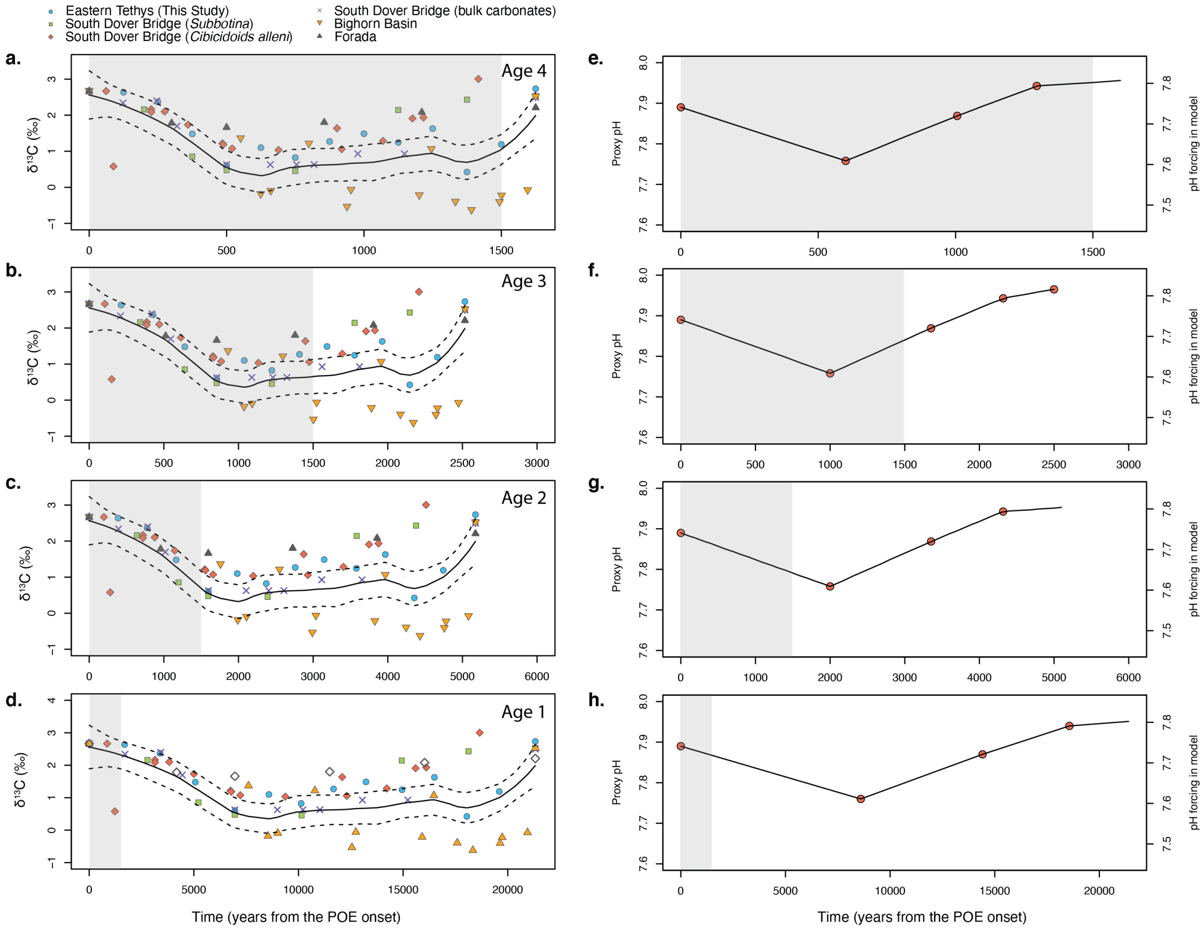

Figure S7. Paired "pH– δ^{13}C" forcings used in our "double inversion" experiments using four independent age models. The δ^{13}C data are from a global compilation (blue circles are from the eastern Tethys (this study), green circles are from benthic foraminifera *Subbotina* at South Dover Bridge[34], red circles are from benthic foraminifera *Cibicidoids alleni* at South Dover Bridge[34], black cross represents bulk carbonate from South Dover Bridge[34], yellow upsidedown trianbles are from pedogenic carbonate from Wyoming Bighorn Basin[35], and blue triangles are bulk carbonate from Forada, Italy[36]). The pH data are from benthic foraminifera *Cibicidoids alleni* at South Dover Bridge[34]. The magnitude of pH change used here is a conservative estimate of ~0.1 unit decrease from the onset of the POE. (a-d) Observed δ^{13}C data and LOESS (local regression) fit *versus* four independent age models. (e-h) Proxy-derived surface ocean pH and the pH forcing used in our model simulations *versus* four independent age models.

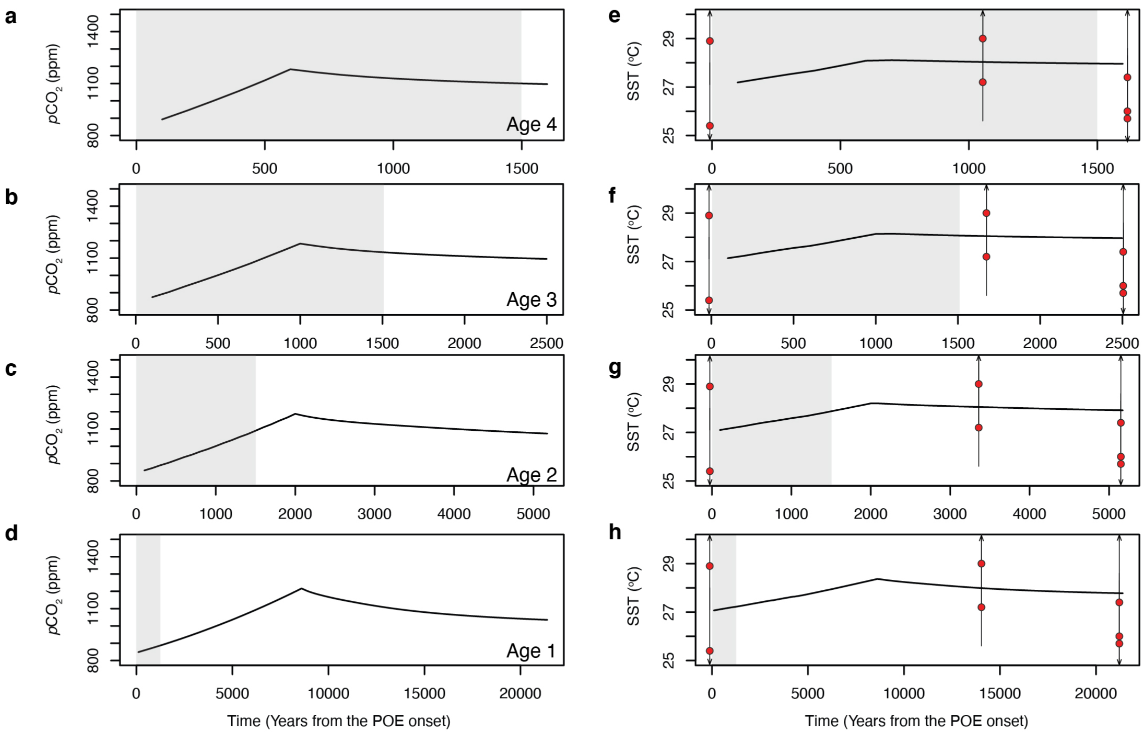

Figure S8. cGENIE model results of atmospheric pCO_2 and sea surface temperature (SST) for the four independent age models. (a-d) Simulated atmospheric pCO_2. (e-h) Simulated global SST changes in response to thermogenic CO_2 emissions during the POE. The red circles and vertical bars represent sea surface temperature reconstructed from Mg/Ca ratios of foraminifera from the Mid-Atlantic Coastal Plain[34].

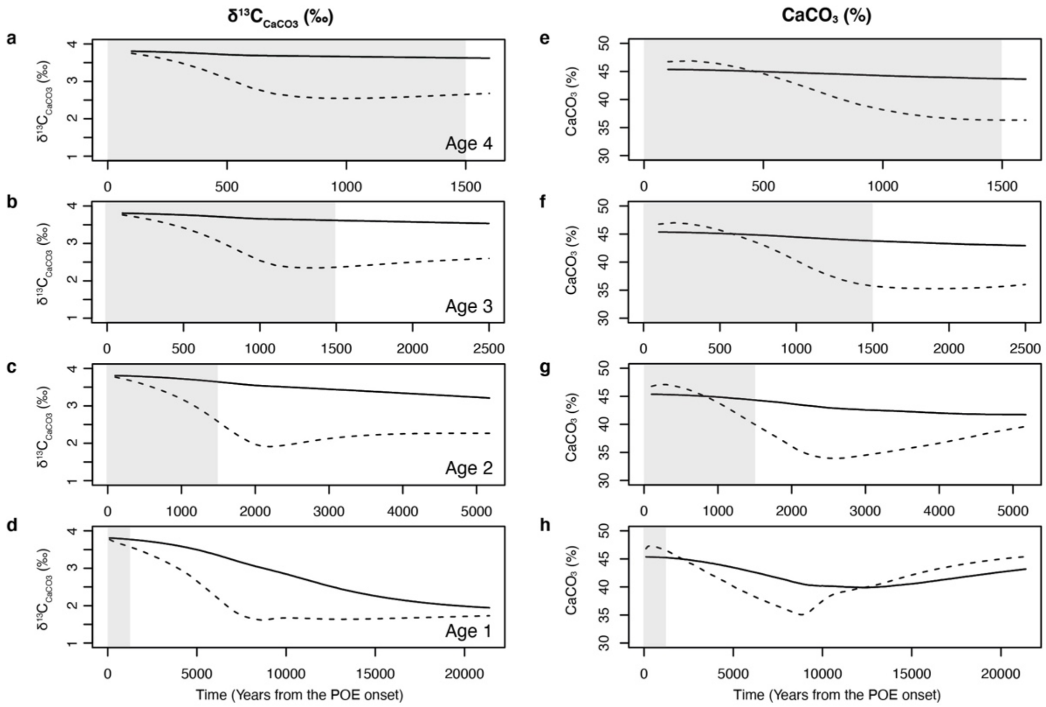

Figure S9. cGENIE model results of bioturbation sensitivity experiments for the POE using four independent age models. **a-d**. Core-top $\delta^{13}C$ of carbonate for experiments with bioturbation on (solid lines) and off (dashed lines) varying the experiment duration using four independent age models. **e-h.** Core-top carbonate wt.% for experiments with bioturbation on (solid lines) and off (dashed lines) varying the experiment duration using four independent age models.

Table S1. **Key results from individual model runs for the POE.**

Experiment ID	Experimental assumptions				Peak emissions (Pg C yr^{-1})	Cumulative emissions (Pg C)	Mean flux-weighted $\delta^{13}C_{source}$ (‰)	ΔpCO2 (µatm)	ΔSST (°C)
	ΔpH	Duration of the POE onset (years)	$\delta^{13}C$ forcing	Age option					
1	0.08	~7,000	Global compilation	Age 1	0.4	1765	−43.9	383	1.3
2	0.08	~1,600		Age 2	0.8	1238	−44.5	354	1.2
3	0.08	~850		Age 3	1.3	1105	−37.2	350	1.1
4	0.08	~500		Age 4	2.0	1026	−30.8	349	1.1
Sensitivity 1	0.24	~7,000		Age 1	0.6	3486	−24.4	810	2.5
Sensitivity 2	0.24	~1,600		Age 2	1.6	2372	−24.7	729	2.2
Sensitivity 3	0.24	~850		Age 3	2.7	2137	−21.1	720	2.1
Sensitivity 4	0.24	~500		Age 4	4.2	2006	−19.4	719	2.1
Sensitivity 5	0.32	~7,000		Age 1	0.9	4940	−17.3	1222	3.4
Sensitivity 6	0.32	~1,600		Age 2	2.4	3394	−18.3	1093	3.0
Sensitivity 7	0.32	~850		Age 3	4.2	3076	−15.9	1080	2.9
Sensitivity 8	0.32	~500		Age 4	6.4	2901	−14.9	1073	2.8

*Peak excess weathering refers to the difference between silicate weathering that leads to CO_2 removal from the atmosphere and the pre-POE weathering rates. pH option 1 refers to the assumed initial surface ocean pH of 7.89 and pH option 2 refers to the assumed initial surface ocean pH of 7.67 based on Babila et al.[34] Note that initial pH used in the "double inversion" has been normalized to 7.741 to be consistent with the cGENIE open-system spinup surface pH value.

Supplementary References

1. Yin A*, et al.* Tectonic history of the Altyn Tagh fault system in northern Tibet inferred from Cenozoic sedimentation. *GSA Bulletin* **114**, 1257-1295 (2002).

2. Wang QM, Nishidai T, Coward MP. The Tarim Basin, NW China: Formation and aspects of petroleum geology *Journal of Petroleum Geology* **15**, 5-34 (1992).

3. Jia C, Wei G, Wang L. *Structural characteristics and oil and gas in the Tarim Basin, China (in Chinese)*. Petroleum Industry Press (1997).

4. Sciences CAoG. *Chinese stratigraphy. 1. Introduction to Chinese Stratigraphy (in Chinese)*. Geological Press (1982).

5. Bosboom R*, et al.* Linking Tarim Basin sea retreat (west China) and Asian aridification in the late Eocene. *Basin Research* **26**, 621-640 (2014).

6. Chai G, Wang X, Jin X. Petroleum Geology and Oil Potential of Tarim Basin, West China. In: *13th World Petroleum Congress*). World Petroleum Congress (1991).

7. Carroll AR, Graham SA, Hendrix MS, Ying D, Zhou D. Late Paleozoic tectonic amalgamation of northwestern China: Sedimentary record of the northern Tarim, northwestern Turpan, and southern Junggar Basins. *GSA Bulletin* **107**, 571-594 (1995).

8. He D, Jia C, Li D, Zhang C, Meng Q, Shi X. Formation and evolution of polycyclic superimposed Tarim Basin. *Oil & Gas Geology* **26**, 64-77 (2005).

9. Hendrix MS*, et al.* Sedimentary record and climatic implications of recurrent deformation in the Tian Shan: Evidence from Mesozoic strata of the north Tarim, south Junggar, and Turpan basins, northwest China. *GSA Bulletin* **104**, 53-79 (1992).

10. Tang T. *Late Cretaceous to Early Paleogene sedimentary characteristics and depositional environment of the western Tarim Basin in Xinjiang (in Chinese)*. Science Press (1992).

11. Burtman VS. Cenozoic crustal shortening between the Pamir and Tien Shan and a reconstruction of the Pamir–Tien Shan transition zone for the Cretaceous and Palaeogene. *Tectonophysics* **319**, 69-92 (2000).

12. Zhong S. Calcareous nannofossils from the Cretaceous Kukebai Formation in the western Tarim Basin, South Xinjiang, China (in Chinese with English abstract). *Acta Micropalaeontologica Sinica* **1**, 201-224 (1984).

13. Zhang S, Hu X, Han Z, Li J, Garzanti E. Climatic and tectonic controls on Cretaceous-Palaeogene sea-level changes recorded in the Tarim epicontinental sea. *Palaeogeography, Palaeoclimatology, Palaeoecology* **501**, 92-110 (2018).

14. Mao S, Norris G. Late Cretaceous-Early Tertiary dinoflagellates and acritarchs in the western Tarim Basin, Xinjiang (in Chinese with English abstract). *Earth Science* **7**, 139-141 (1984).

15. Bosboom R*, et al.* Late Eocene sea retreat from the Tarim Basin (west China) and concomitant Asian paleoenvironmental change. *Palaeogeography, Palaeoclimatology, Palaeoecology* **299**, 385-398 (2011).

16. Zhang Z, Wang H, Guo Z, Jiang D. What triggers the transition of palaeoenvironmental patterns in China, the Tibetan Plateau uplift or the Paratethys Sea retreat? *Palaeogeography, Palaeoclimatology, Palaeoecology* **245**, 317-331 (2007).

17. Ramstein G, Fluteau F, Besse J, Joussaume S. Effect of orogeny, plate motion and land–sea distribution on Eurasian climate change over the past 30 million years. *Nature* **386**, 788-795 (1997).

18. Zhu J, Poulsen CJ, Tierney JE. Simulation of Eocene extreme warmth and high climate sensitivity through cloud feedbacks. *Science Advances* **5**, eaax1874 (2019).

19. Bosboom R, Mandic O, Dupont-Nivet G, Proust J, Ormukov C, Aminov J. Late Eocene palaeogeography of the proto-Paratethys Sea in Central Asia (NW China, southern Kyrgyzstan and SW Tajikistan). *Geological Society, London, Special Publications* **427**, 565 (2015).

20. Jiang J*, et al.* Eustatic change across the Paleocene-Eocene Thermal Maximum in the epicontinental Tarim seaway. *Global and Planetary Change* **229**, 104241 (2023).

21. Jiang T, Wan X, Aitchison JC, Xi D, Cao W. Foraminiferal response to the PETM recorded in the SW Tarim Basin, central Asia. *Palaeogeography, Palaeoclimatology, Palaeoecology* **506**, 217-225 (2018).

22. Lippert PC, van Hinsbergen DJJ, Dupont-Nivet G. Early Cretaceous to present latitude of the central proto-Tibetan Plateau: A paleomagnetic synthesis with implications for Cenozoic tectonics, paleogeography, and climate of Asia. In: *Toward an Improved Understanding of Uplift Mechanisms and the Elevation History of the Tibetan Plateau* (eds Nie J, Horton BK, Hoke GD). Geological Society of America (2014).

23. Knauth LP, Kennedy MJ. The late Precambrian greening of the Earth. *Nature* **460**, 728-732 (2009).

24. Wang Y*, et al.* Response of calcareous nannoplankton to the Paleocene–Eocene Thermal Maximum in the Paratethys Seaway (Tarim Basin, West China). *Global and Planetary Change*, 103918 (2022).

25. Westerhold T*, et al.* Synchronizing early Eocene deep-sea and continental records–new cyclostratigraphic age models from the Bighorn Basin Coring Project. (2018).

26. Röhl U, Westerhold T, Bralower TJ, Zachos JC. On the duration of the Paleocene-Eocene thermal maximum (PETM). *Geochemistry, Geophysics, Geosystems* **8**, Q12002 (2007).

27. Zeebe RE, Lourens LJ. Solar System chaos and the Paleocene–Eocene boundary age constrained by geology and astronomy. *Science* **365**, 926-929 (2019).

28. Charles AJ*, et al.* Constraints on the numerical age of the Paleocene-Eocene boundary. *Geochem Geophys Geosyst* **12**, Q0AA17 (2011).

29. Farley KA, Eltgroth SF. An alternative age model for the Paleocene–Eocene thermal maximum using extraterrestrial 3He. *Earth and Planetary Science Letters* **208**, 135-148 (2003).

30. Murphy BH, Farley KA, Zachos JC. An extraterrestrial 3He-based timescale for the Paleocene-Eocene thermal maximum (PETM) from Walvis Ridge, IODP Site 1266. *Geochimica et Cosmochimica Acta* **74**, 5098-5108 (2010).

31. Meyers SR. The evaluation of eccentricity ‐ related amplitude modulation and bundling in paleoclimate data: An inverse approach for astrochronologic testing and time scale optimization. *Paleoceanography* **30**, 1625-1640 (2015).

32. Li M, Hinnov L, Kump L. Acycle: Time-series analysis software for paleoclimate research and education. *Computers & Geosciences* **127**, 12-22 (2019).

33. Kender S*, et al.* Paleocene/Eocene carbon feedbacks triggered by volcanic activity. *Nature Communications* **12**, 1-10 (2021).

34. Babila TL*, et al.* Surface ocean warming and acidification driven by rapid carbon release precedes Paleocene-Eocene Thermal Maximum. *Science Advances* **8**, eabg1025 (2022).

35. Bowen GJ*, et al.* Two massive, rapid releases of carbon during the onset of the Palaeocene–Eocene thermal maximum. *Nature Geoscience* **8**, 44-47 (2015).

36. Giusberti L*, et al.* Mode and tempo of the Paleocene-Eocene Thermal Maximum in an expanded section from the Venetian pre-Alps. *Geological Society of America Bulletin* **119**, 391-412 (2007).
