# Supplementary material for: Millennial-timescale thermogenic CO2 release preceding the Paleocene-Eocene Thermal Maximum
Source: Nat Commun. 2025 Jun 30;16:5375. doi: 10.1038/s41467-025-60939-3 (PMC12209464; doi:10.1038/s41467-025-60939-3)
Supplement: Supplementary file 2 — Description of Additional Supplementary Files [file 41467_2025_60939_MOESM2_ESM.pdf]

Description of Additional Supplementary Files:

Supplementary Data 1: Data for XRF elements, bulk carbonate isotopes, bulk organic carbon isotopes, mercury content, long-chain n-alkane $\delta^{13}C$, apolar biomarkers, GDGTs, and clumped isotopes.

Supplementary Data 2: Astronomical age models.
